# The Effect of Preoperative Biliary and Pancreatic Drainage on Postoperative Pancreatic Fistula: A Retrospective Cohort Study

**DOI:** 10.1055/s-0038-1639343

**Published:** 2018-03-20

**Authors:** John Mathew Manipadam, Mahesh S., Jacob Mathew Kadamapuzha, Ramesh H.

**Affiliations:** 1 Department of GI Surgery and Liver Transplantation, VPS Lakeshore Hospital and Research Centre, Kochi, Kerala, India

**Keywords:** preoperative biliary drainage, postoperative pancreatic fistula, pancreaticoduodenectomy

## Abstract

**Background** Surgeons and endoscopists welcome routine preoperative biliary drainage prior to pancreaticoduodenectomy despite evidence that it increases complications. Its effect on postoperative pancreatic fistula is variably reported in literature. Simultaneous biliary and pancreatic drainage is rarely performed for very selected indications and its effects on postoperative pancreatic fistula are largely unknown. Our aim was to analyze the same while eliminating confounding factors.

**Methods** Retrospective single center cohort study of patients who underwent pancreaticoduodenectomy over the past 10 years for carcinoma obstructing the lower common bile duct. Patients who underwent biliary stenting alone, biliary and pancreatic stenting, and no stenting prior to pancreaticoduodenectomy were the three study cohort groups and their records were scrutinized for the incidence of postoperative pancreatic fistula.

**Results** Sixty-two patients underwent biliary stenting alone, 5 patients underwent both biliary and pancreatic stenting, and 237 patients were not stented in the adenocarcinoma group without chronic pancreatitis. The pancreatic fistula rate was similar in the patients who underwent biliary stenting alone when compared with the group which was not stented. (24/62 versus 67/237, odds ratio [OR] =0.619, confidence interval (CI) =0.345–1.112, *p* = 0.121). However, the patients who underwent both biliary and pancreatic stenting had a significant increase in postoperative pancreatic fistula compared with the not stented ( *p* = 0.003). By univariate and multivariate analysis using Firth logistic regression, pancreatic texture (OR = 1.205, CI = 0.103–2.476, *p* = 0.032) and the presence of a biliary and pancreatic stent (OR = 2.695, CI = 0.273–7.617, *p* = 0.027) were the significant factors affecting pancreatic fistula.

**Conclusion** Preoperative biliary drainage alone has no significant influence on postoperative pancreatic fistula except when combined with pancreatic stenting. We need more such studies from other centers to confirm that the rare event of preoperative biliary and pancreatic stenting has indeed this harmful effect on healing of postoperative pancreatic anastomosis.

The effect of preoperative biliary drainage (PBD) prior to pancreaticoduodenectomy (PD) on postoperative pancreatic fistula (POPF) is variably reported in literature. Initial studies on PBD included both benign and malignant pathologies, employed the percutaneous means of biliary drainage, and did not specifically address POPF after PD. 1 2 3 4 5 6 7 Subsequently, some centers reported increased POPF rates after PD with PBD. 8 9 10 11 However, large series spoke of no such increase. 12 13 14 Although several meta-analyses and other retrospective studies have examined stented versus nonstented patients, they have not specifically addressed POPF rates. 15 16 17 18 19 20 21 22 23 Preoperative pancreatic drainage is rarely performed for very selected indications such as preoperative pancreatitis, after initial endoscopic ampullectomy, and its effects on POPF are largely unknown.

Why should biliary or pancreatic stenting affect the incidence of POPF? Stent placement can not only induce pancreatic and bile duct wall inflammation but also introduce infection into the biliopancreatic system, and this may be responsible. 24 25 Does the addition of pancreatic stenting contribute to a higher morbidity after PD than biliary stenting alone? However, the increase in POPF rates after stenting may well be as a result of other confounding factors such as disease stage, pancreatic texture, and ductal diameter. 8 26 To address this issue, a retrospective cohort analysis of data over a 10-year period was performed.

## Aims

1. To analyze the incidence of POPF in patients who underwent biliary stenting versus those who were not stented prior to PD and whether it affected the duration of hospital stay.
2. To analyze whether the addition of pancreatic stenting adds to the incidence of POPF.
3. To determine whether demographic, preoperative, and intraoperative parameters have significantly affected the incidence of pancreatic fistula in these groups.

## Methods

### Patient Eligibility Criteria

A retrospective observational longitudinal cohort study was performed after extracting the data of patients who underwent PD over the past 10 years from the prospectively maintained database in this center. Approval from the institutional reviewer board and ethics committee was obtained for conducting this study and the approval number is LEC/DMS/T/001–17.

### Inclusion Criteria

Patients with carcinoma causing obstruction to the lower end of bile duct without chronic pancreatitis who underwent PD.

### Exclusion Criteria

Patients who had undergone prior surgical bypass: The patients who underwent biliary stenting alone represented group 1, those who underwent biliary and pancreatic stenting were put in group 2, and those not stented were assigned in group 3. Sequential consecutive sampling was used. Pancreatic fistula in the postoperative period was defined according to the ISGPF criteria as persistent drainage of fluid on or after postoperative day 3 with an amylase content greater than three times the upper normal serum value. The indication for stenting in these patients was determined by examining the medical records. The demographics, preoperative, intraoperative parameters, and postoperative outcome were recorded.

### Data Collection

The variables are shown in Table 1 . These were recorded and compared for group 1, group 2, and group 3. For bilirubin values, prestenting bilirubin in group 1 and 2 and preoperative bilirubin in group 3 were recorded and compared by dividing them further into three subgroups based on the levels of bilirubin (0–10, 10–20, and ≥ 20 mgs%).

**Table 1:** Variables analyzed in the study

Preoperative	Intraoperative	Postoperative
Age	Pancreatic duct diameter (millimeters)	Pancreatic fistula
Sex	Pancreatic texture (soft, firm, or hard)	Postpancreatectomy hemorrhage
Co-morbidities	Requirement for portal vein resection	Duration of hospital stay
Serum albumin		
Serum preoperative/prestenting bilirubin		

Hospital stay was taken as the duration from the date of operation till the day of discharge. All these details were recorded and tabulated in Microsoft Excel for each of the patients.

Prior to operation, patients underwent a routine preoperative workup to assess fitness and a CECT abdomen to assess resectability. Endoscopic biopsy was performed in all periampullary tumors. Endoscopic stenting was universally done with a plastic stent in resectable lesions. At operation, a standard pylorus-resecting PD was performed in all the cases with a duct to mucosa pancreaticojejunostomy.

### Primary Outcome

Primary outcome is the incidence of POPF and duration of hospital stay in the stented versus the nonstented patients who underwent PD in the past 10 years at our institution.

### Secondary Outcome

Secondary outcome is the association of demographic, preoperative, and intraoperative variables with the incidence of pancreatic fistula.

### Statistical Analysis

Statistical analysis was performed using Microsoft Excel and Graph Pad Prism with the help of a statistician. Categorical variables were expressed as frequencies and analyzed using Fishers exact test, while continuous variables were expressed as median (interquartile range [IQR]) and analyzed using Mann–Whitney U test. Univariate and multivariate analysis (Firth logistic regression) for predictive factors of pancreatic fistula was performed using SPSS Version 24. Firth logistic regression was used to reduce the bias of binary logistic regression in the analysis of rare events (biliary and pancreatic stent) by using a penalized maximum likelihood estimation. 27 28 29 Factors with *p* value < 0.20 on univariate analyses were included in the multivariate analyses. Patients with missing values were excluded from the analysis.

### Patients

Three-hundred and ten patients with carcinoma obstructing the lower end of common bile duct (CBD) were identified who underwent PD. Six were excluded because they underwent surgical biliary bypass prior to PD. One-hundred and seventy-nine patients had periampullary, 96 had tumor located in the head of the pancreas, 18 and 11 had cholangiocarcinomas and duodenal adenocarcinomas respectively. There were 62 patients who underwent biliary stenting alone, 5 who underwent both biliary and pancreatic stenting, and 237 patients who were not stented, but underwent direct surgery. The groups were comparable for all parameters including subtype of carcinoma, except for albumin levels which were marginally lower in the stented group ( Table 2 ).

**Table 2:** Comparison of preoperative and intraoperative parameters in stented versus not stented patients

Parameter a	Not stented(237)	Stented (67)	*p* Value
Age	60(53–65)	60(49–69)	0.501
Males	143	44	0.479
Comorbidities	105	27	0.580
Preoperative albumin	3.8(3.5–4.1)	3.5(3.2–3.9)	0.008
Firm/hard pancreas	44	14	0.725
Pancreatic duct diameter(mm)	5(3–5.5)	4(3–6.75)	0.495
Distribution of type of adenocarcinoma(periampullary/other types)	133/104	34/33	0.219

a Median with interquartile range for continuous variable.

## Results

**Fig. 1:**
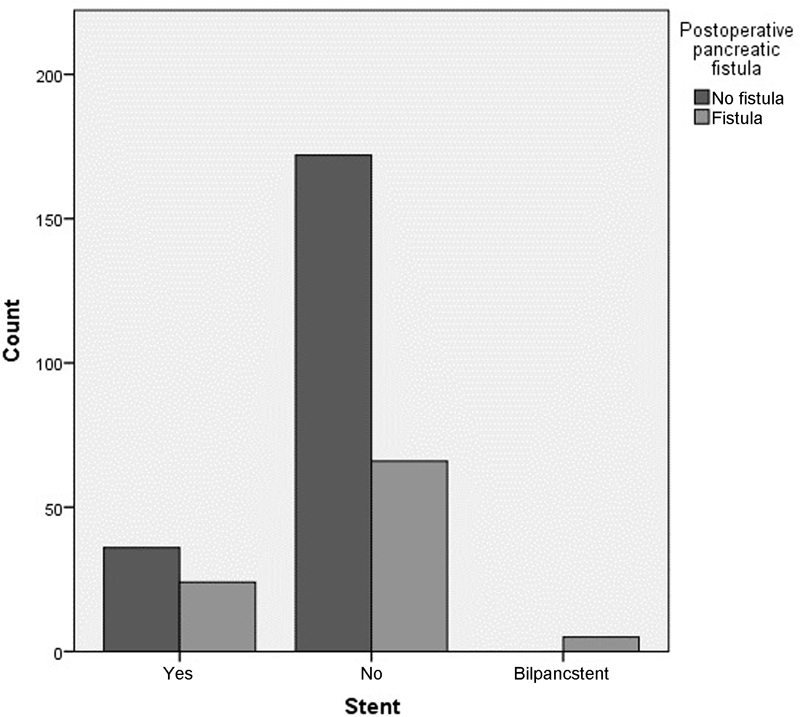
Postoperative pancreatic fistula in stented versus nonstented bilpancstent—biliary and pancreatic stent.

POPF developed in 24 out of 62 (38.7%) patients in the group1, 5 out of 5 (100%) patients in group 2, and in 67 out of 237 (28.3%) patients in the nonstented group. There was no statistically significant difference in pancreatic fistula rates in group 1 versus group 3 (odds ratio [OR] =0.619, confidence interval [CI] =0.345–1.112, *p* = 0.121). However, there was a statistically significant increase in the pancreatic fistula rates in the patients who underwent biliary and pancreatic stenting (group 2) compared with the nonstented (group 3) ( *p* = 0.003) ( Fig. 1 ).

There was no significant difference in the duration of hospital stay in stented (median [IQR] = 15 days [13–21]) versus nonstented (median [IQR] = 14 days [11–19]) patients. ( *p* = 0.09).

From the medical records, the reason for stenting was probed and it showed that documented cholangitis was seen in only six patients. The small number of patients who underwent both biliary and pancreatic stenting had it done after endoscopic ampullectomy and one for presentation of pancreatitis with periampullary malignancy.

Biliary stenting was not associated with any significant difference in the pancreatic fistula rates across all the bilirubin subgroups ( Table 3 ). Univariate analysis also pointed out that serum bilirubin level does not affect the pancreatic fistula rate. Stenting did not affect the rate of postpancreatectomy hemorrhage either in all the bilirubin subgroups ( Table 3 ).

**Table 3:** Subgroup analysis of the effect of preoperative and prestenting serum bilirubin on POPF and PPH

Preoperative/prestenting bilirubin a	Not stented				Stented				*p* Value	
	POPF	No POPF	PPH	No PPH	POPF	No POPF	PPH	No PPH	POPF stented versus not stented	PPH stented versus not stented
< 10	45	122	13	154	5	10	2	13	0.560	0.356
10–20	9	37	5	41	5	6	1	10	0.116	1.000
≥ 20	4	6	0	10	2	7	0	9	0.629	1.000

Abbreviations: POPF, postoperative pancreatic fistula rates; PPH, postpancreatectomy hemorrhage.

a 41 bilirubin values missing, hence only 263 analyzed.

On univariate analysis of the predictive factors, there was a significant association of six factors, namely age, serum albumin, biliary and pancreatic stenting, portal vein infiltration, pancreatic duct diameter, and pancreatic texture, with POPF. However, on multivariate analysis (Firth logistic regression after selecting those variables with *p* value < 0.20 on univariate analysis), pancreatic texture and the presence of a biliary and pancreatic stent were the only two factors that were significantly affecting the pancreatic fistula rate ( Table 4 ).

**Table 4:** Univariate and multivariate analysis of predictive factors for occurrence of POPF

Factor		Total (304)	Univariate analysis		Multivariate analysis	
			*p* Value	OR	*p* Value	OR
Age			0.011	1.033(1.008–1.06)	0.45	1.016(0.974–1.061)
Sex	Male	187	0.151	1.495(0.900–2.517)	0.470	1.412(0.557–3.721)
	Female	117				
Comorbidities	Yes	132	0.874	1.060(0.649–1.726)	NA	
	No	172				
Preoperative bilirubin			0.802	1.003 (0.967–1.043)	NA	
Preoperative serum albumin			0.041	1.833(1.036–3.418)	0.496	1.335(0.598–3.360)
Biliary and pancreatic stenting			0.002	25.33(2.826–3340.915)	0.027	14.806(1.314–2032.46)
Biliary Stent	Yes	62	0.108	1.642(0.910- 2.930)	0.118	2.291(0.808–6.666)
	No	237				
Portal vein infiltration	Yes	17	0.011	5.700(1.404–52.100)	0.831	1.237(0.771-.1.851)
	No	287				
Pancreatic duct diameter			0.002	1.266(1.081–1.511)	0.508	1.077(0.870–1.365)
Pancreatic texture	Firm/Hard	83	0.001	2.956(1.484–6.311)	0.032	3.334(1.108–11.89)
	Soft	221				

Abbreviation: NA, not available; OR, odds ratio; POPF, postoperative pancreatic fistula.

## Discussion

There is a division of opinion in relevant literature as regards the effect of preoperative stenting on POPF. In 1998, Povoski et al from MSKCC retrospectively analyzed 240 PD and clearly showed that PBD is associated with an increased incidence of postoperative complications, infectious complications, intra-abdominal abscesses, and death. 12 However, surgical, endoscopic, and percutaneous biliary drainage procedures were included in this analysis unlike ours where we have excluded surgical PBD.

In 2000, Sohn et al reported from an analysis of 567 patients that preoperative biliary stenting prior to PD does increase the rate of pancreatic fistula formation and wound infection; however, it does not affect the overall morbidity or mortality. 8 However, 64% of the patients were stented via a percutaneous approach. A recent retrospective analysis from Nagoya University also confirmed that endoscopic stenting of the CBD is an independent predictor of POPF after PD. 11

Pisters et al from MD Anderson Cancer Center concluded on the contrary from an analysis of 300 patients that preoperative biliary stenting prior to PD does not increase the rate of major postoperative complications or mortality except for wound infection. 13 Recent retrospective evidence from large volume centers also concludes that PBD does not affect overall morbidity and mortality of PD except for wound infection 14 The recommendation was, therefore, that patients can be initially treated with endoscopic biliary drainage and need not go for immediate laparotomy.

A well-conducted retrospective study from a single center showed that even in severely jaundiced patients with a bilirubin more than 15 mg/dL, PBD contributed to increased operative time, blood loss, and wound complications without affecting the POPF rate. 30 This is in contrast to some earlier studies that concluded that in severely jaundiced patients, stenting reduces bleeding complications. 9 Thus, we have conflicting results from retrospective studies from different centers.

A very few randomized controlled trials have been done in this topic since it has been proven that PBD definitely increases wound complications. van der Gaag et al conducted a randomized controlled trial comparing plastic stenting versus direct surgery for cancers of the head of pancreas with a maximum bilirubin value of 14.6, which were not locally advanced and not in cholangitis. 31 This is the only randomized controlled trial which has specifically addressed PBD by endoscopic stenting prior to PD. The occurrence of stent-related complications in significant numbers led to the conclusion that routine PBD is not advisable. One drawback of this study is that it does not analyze pathologies other than carcinoma head of pancreas where the pancreatic fistula rates are known to be different. Also, patients with a bilirubin of 15 or more and those with significant portal vein invasion are excluded from this study.

Due to the paucity of randomized controlled trials, we can rely only on retrospective data and meta-analyses. However, recent review articles and meta-analyses on this topic have also come out with conflicting results. Moole et al concluded that PBD reduces morbidity after PD, 32 while Lai et al advocated that it does not have any beneficial effect on periampullary tumors. 33 A very few such as Chen et al have specifically addressed POPF rate which is a significant factor affecting the postoperative course. 34 Most of the other meta-analyses have not been able to shed light on this subject. 15 16 17 18 19 20 21 22 23 The probable reason for such conflicting results in these meta-analyses is the inclusion of heterogeneous studies, such as including randomized controlled trials which studied proximal as well as distal bile duct malignancies, endoscopic as well as percutaneous biliary drainage, bypass and palliative resections. 33

Thus, the importance and clarity of single center retrospective studies emerge where patients are operated by a single surgical team with a standardized procedure, thereby eliminating bias that creeps into clubbing heterogeneous studies. 30 In addition to being a single center study, we have selectively chosen only adenocarcinomas obstructing the lower CBD, thereby eliminating bias that can be brought in by different pathologies that are known to affect POPF such as underlying chronic pancreatitis, intraductal papillary mucinous neoplasm (IPMN), neuroendocrine tumors, and cystic neoplasms.

A limitation of this study seems to be the fact that there are earlier randomised trials and meta-analyses published on PBD. Another limitation is that there can be several confounding factors influencing the primary outcome of this study which is POPF such as pancreatic texture, pancreatic duct diameter, comorbidities, age, sex, serum albumin, serum bilirubin, and portal vein infiltration. However, we have negated this bias by a multivariate analysis which included all these confounding factors. The other limitation is that the sample size in the stented group is much less. Again there is a definite reason for lower size of the stented group, since evidence is already established that PBD increases septic wound complications, leading us away from routine to selective PBD. Many patients who have been referred to us with a stent already placed have contributed to this number of 67. On the whole, the number of 304 patients was adequate sample size for this cohort study. This is proven by the fact that the confidence intervals of the significant results are not crossing one. Also we have selected only adenocarcinomas obstructing the lower CBD, thereby eliminating bias that could be brought out by heterogeneous pathologies such as underlying chronic pancreatitis, IPMN, neuroendocrine tumors, and cystic neoplasms.

Simultaneous biliary and pancreatic stenting is rarely done and so the sample will always be small. Prospective studies cannot also be done in this topic for the same reason. So, a retrospective multivariate analysis adjusting for rare events using established statistical methods such as Firth logistic regression seems appropriate.

Analysis of our data showed that biliary stenting alone does not significantly affect the incidence of pancreatic fistula across all the bilirubin subgroups except when combined with pancreatic stenting. This is probably because of pancreatic duct wall inflammation at the site of the future anastomosis and bacterial infection of the bile and pancreatic fluid induced by stenting as has been reported before. 24 25 The result we obtained adds evidence to the hypothesis that pancreatic ductal wall inflammation induced by the pancreatic stent at the site of the future pancreaticoenteral anastomosis may be responsible for POPF and that biliary stent per se does not have any adverse effect at this site. In other words, careful biliary cannulation avoiding repeated inadvertent pancreatic duct cannulation is of utmost importance. This would reduce the need for prophylactic pancreatic duct stenting to prevent pancreatitis.

Pancreatic stenting may reduce the incidence of postendoscopic retrograde cholangiopancreatography (ERCP) pancreatitis in difficult biliary cannulation and after endoscopic ampullectomy. 35 However, it has its limitations such as unsuccessful stent placement due to the inability to advance a wire into the PD or the inability to place a stent after wire placement. This results in an increased risk of post-ERCP pancreatitis. 36 There can also be inadvertent duct injury during stent placement and long-term stent-related duct or gland injury. Variable expertise and familiarity with their placement in less experienced hands are indeed a point against prophylactic pancreatic stenting. 36 We have found in our study that the addition of pancreatic to biliary stenting may increase the rate of POPF significantly in the postoperative period. So, our recommendation is to endorse PBD in selected patients prior to PD, however, with utmost care and technique to avoid repeated pancreatic cannulation and the need for a pancreatic stent. More studies from other centers are required to confirm the same as combined biliary and pancreatic stenting is rarely done. Yet it assumes significance if it has a harmful effect on healing of the pancreatic anastomosis as has been proven in our study.

## Conclusion

PBD alone has no significant effect on POPF except when combined with pancreatic stenting.

## References

[1] Dixon J M Armstrong C P Duffy S W Davies G C Factors affecting morbidity and mortality after surgery for obstructive jaundice: a review of 373 patients Gut 1983 24 09 845 852 6604001 10.1136/gut.24.9.845 PMC1420091

[2] Denning D A Ellison E C Carey L C Preoperative percutaneous transhepatic biliary decompression lowers operative morbidity in patients with obstructive jaundice Am J Surg 1981 141 01 61 65 6779653 10.1016/0002-9610(81)90013-1

[3] Gobien R P Stanley J H Soucek C D Anderson M C Vujic I Gobien B S Routine preoperative biliary drainage: effect on management of obstructive jaundice Radiology 1984 152 02 353 356 6739798 10.1148/radiology.152.2.6739798

[4] Gundry S R Strodel W E Knol J A Eckhauser F E Thompson N W Efficacy of preoperative biliary tract decompression in patients with obstructive jaundice Arch Surg 1984 119 06 703 708 6428380 10.1001/archsurg.1984.01390180065011

[5] Hatfield A R Tobias R Terblanche J Preoperative external biliary drainage in obstructive jaundice. A prospective controlled clinical trial Lancet 1982 2(8304):896 899 6126752 10.1016/s0140-6736(82)90866-2

[6] McPherson G A Benjamin I S Hodgson H J Bowley N B Allison D J Blumgart L H Pre-operative percutaneous transhepatic biliary drainage: the results of a controlled trial Br J Surg 1984 71 05 371 375 6372935 10.1002/bjs.1800710522

[7] Pitt H A Gomes A S Lois J F Mann L L Deutsch L S Longmire W P Jr Does preoperative percutaneous biliary drainage reduce operative risk or increase hospital cost? Ann Surg 1985 201 05 545 553 2986562 10.1097/00000658-198505000-00002 PMC1250755

[8] Sohn T A Yeo C J Cameron J L Pitt H A Lillemoe K D Do preoperative biliary stents increase postpancreaticoduodenectomy complications? J Gastrointest Surg 2000 4 03 258 267, discussion 267–268 10769088 10.1016/s1091-255x(00)80074-8

[9] Srivastava S Sikora S S Kumar A Saxena R Kapoor V K Outcome following pancreaticoduodenectomy in patients undergoing preoperative biliary drainage Dig Surg 2001 18 05 381 387 11721113 10.1159/000050178

[10] Morris-Stiff G Tamijmarane A Tan Y-M Pre-operative stenting is associated with a higher prevalence of post-operative complications following pancreatoduodenectomy Int J Surg 2011 9 02 145 149 21029795 10.1016/j.ijsu.2010.10.008

[11] Fujii T Yamada S Suenaga M Preoperative internal biliary drainage increases the risk of bile juice infection and pancreatic fistula after pancreatoduodenectomy: a prospective observational study Pancreas 2015 44 03 465 470 25423556 10.1097/MPA.0000000000000265

[12] Povoski S P Karpeh M SJ Conlon K C Blumgart L H Brennan M F Association of preoperative biliary drainage with postoperative outcome following pancreaticoduodenectomyAnn Surg 1999;230(2) 10.1097/00000658-199908000-00001 PMC1420854 10450725

[13] Pisters P W Hudec W A Hess K R Effect of preoperative biliary decompression on pancreaticoduodenectomy-associated morbidity in 300 consecutive patients Ann Surg 2001 234 01 47 55 11420482 10.1097/00000658-200107000-00008 PMC1421947

[14] Sahora K Morales-Oyarvide V Ferrone C Preoperative biliary drainage does not increase major complications in pancreaticoduodenectomy: a large single center experience from the Massachusetts General Hospital J Hepatobiliary Pancreat Sci 2016 23 03 181 187 26768943 10.1002/jhbp.322

[15] Singhirunnusorn J Roger L Chopin-Laly X Lepilliez V Ponchon T Adham M Value of preoperative biliary drainage in a consecutive series of resectable periampullary lesions: from randomized studies to real medical practice Langenbecks Arch Surg 2013 398 02 295 302 23007383 10.1007/s00423-012-1000-2

[16] Heslin M J Brooks A D Hochwald S N Harrison L E Blumgart L H Brennan M F A preoperative biliary stent is associated with increased complications after pancreatoduodenectomy Arch Surg 1998 133 02 149 154 9484726 10.1001/archsurg.133.2.149

[17] Temudom T Sarr M G Douglas M G Farnell M B An argument against routine percutaneous biopsy, ERCP, or biliary stent placement in patients with clinically resectable periampullary masses: a surgical perspective Pancreas 1995 11 03 283 288 8577683 10.1097/00006676-199510000-00011

[18] Chen D Liang L-J Peng B-G [Effect of preoperative biliary drainage on liver function changes in patients with malignant obstructive jaundice in the low bile duct before and after pancreaticoduodenectomy]. Ai Zheng Aizheng Chin J Cancer 2008 27 01 78 82 18184470

[19] Sewnath M E Karsten T M Prins M H Rauws E JA Obertop H Gouma D J A meta-analysis on the efficacy of preoperative biliary drainage for tumors causing obstructive jaundice Ann Surg 2002 236 01 17 27 12131081 10.1097/00000658-200207000-00005 PMC1422544

[20] Qiu Y-D Bai J-L Xu F-G Ding Y-T Effect of preoperative biliary drainage on malignant obstructive jaundice: a meta-analysis World J Gastroenterol 2011 17 03 391 396 21253401 10.3748/wjg.v17.i3.391 PMC3022302

[21] Fang Y Gurusamy K S Wang Q Meta-analysis of randomized clinical trials on safety and efficacy of biliary drainage before surgery for obstructive jaundice Br J Surg 2013 100 12 1589 1596 24264780 10.1002/bjs.9260

[22] Wang Q Gurusamy K S Lin H Xie X Wang C Preoperative biliary drainage for obstructive jaundice Cochrane Database Syst Rev 2008 03 CD005444 18677779 10.1002/14651858.CD005444.pub2

[23] Fang Y Gurusamy K S Wang Q Pre-operative biliary drainage for obstructive jaundice Cochrane Database Syst Rev 2012 9 09 CD005444 10.1002/14651858.CD005444.pub3 PMC4164472 22972086

[24] Yanagimoto H Satoi S Yamamoto T Clinical impact of preoperative cholangitis after biliary drainage in patients who undergo pancreaticoduodenectomy on postoperative pancreatic fistula Am Surg 2014 80 01 36 42 24401513

[25] Sewnath M E Birjmohun R S Rauws E A Huibregtse K Obertop H Gouma D J The effect of preoperative biliary drainage on postoperative complications after pancreaticoduodenectomy J Am Coll Surg 2001 192 06 726 734 11400966 10.1016/s1072-7515(01)00819-5

[26] Mezhir J J Brennan M F Baser R E A matched case-control study of preoperative biliary drainage in patients with pancreatic adenocarcinoma: routine drainage is not justified J Gastrointest Surg Off J Soc Surg Aliment Tract 2009 13 12 2163 2169 10.1007/s11605-009-1046-9 19774424

[27] Firth D Bias reduction of maximum likelihood estimates Biometrika 1993 80 01 27 38

[28] Heinze G Schemper M A solution to the problem of separation in logistic regression Stat Med 2002 21 16 2409 2419 12210625 10.1002/sim.1047

[29] Roshanov P S Fernandes N Wilczynski J M Features of effective computerised clinical decision support systems: meta-regression of 162 randomised trials BMJ 2013 346 01 f657 23412440 10.1136/bmj.f657

[30] Arkadopoulos N Kyriazi M A Papanikolaou I S Preoperative biliary drainage of severely jaundiced patients increases morbidity of pancreaticoduodenectomy: results of a case-control study World J Surg 2014 38 11 2967 2972 24952079 10.1007/s00268-014-2669-x

[31] van der Gaag N A Rauws E A van Eijck C H Preoperative biliary drainage for cancer of the head of the pancreas N Engl J Med 2010 362 02 129 137 20071702 10.1056/NEJMoa0903230

[32] Moole H Bechtold M Puli S R Efficacy of preoperative biliary drainage in malignant obstructive jaundice: a meta-analysis and systematic reviewWorld Journal of Surgical Oncology [Internet]. 2016 Dec [cited 2018 Jan 8];14(1). Available from:http://wjso.biomedcentral.com/articles/10.1186/s12957-016-0933-2. Accessed February 17, 2018 10.1186/s12957-016-0933-2 PMC4940848 27400651

[33] Lai E CH Lau S HY Lau W Y The current status of preoperative biliary drainage for patients who receive pancreaticoduodenectomy for periampullary carcinoma: a comprehensive review Surgeon 2014 12 05 290 296 24650759 10.1016/j.surge.2014.02.004

[34] Chen Y Ou G Lian G Luo H Huang K Huang Y Effect of preoperative biliary drainage on complications following pancreatoduodenectomy: a meta-analysis Medicine (Baltimore) 2015 94 29 e1199 26200634 10.1097/MD.0000000000001199 PMC4603006

[35] Harewood G C Pochron N L Gostout C J Prospective, randomized, controlled trial of prophylactic pancreatic stent placement for endoscopic snare excision of the duodenal ampulla Gastrointest Endosc 2005 62 03 367 370 16111953 10.1016/j.gie.2005.04.020

[36] Freeman M L Use of prophylactic pancreatic stents for the prevention of post-ERCP pancreatitis Gastroenterol Hepatol (N Y) 2015 11 06 420 422 27118938 PMC4843038

